# A Unique Presentation of Extrapulmonary Legionella: Rhabdomyolysis-Induced Acute Renal Failure and Cerebellar Dysfunction

**DOI:** 10.7759/cureus.28396

**Published:** 2022-08-25

**Authors:** Erik Olson, Minhaz Murshad, Tasnuva Amin, Ndausung Udongwo, Saira Chaughtai, Mohammad A Hossain

**Affiliations:** 1 Internal Medicine, Jersey Shore University Medical Center, Neptune, USA; 2 Medicine, Hackensack Meridian School of Medicine, Nutley, USA

**Keywords:** legionella, cerebellar ataxia, extrapulmonary legionella, neurologic symptoms, dialysis, transaminitis, dysarthria, rhabdomyolysis with acute renal failure, uremic encephalopathy, legionella pneumonia

## Abstract

Legionella is most known for causing pneumonia. However, it is a systemic disease that can directly cause severe multi-organ injury in what is sometimes referred to as "extrapulmonary Legionella." In this case report, a reasonably healthy 80-year-old man is found to have Legionella pneumonia complicated by rhabdomyolysis with acute, severe, non-oliguric acute kidney injury, uremic encephalopathy, transaminitis, and cerebellar dysfunction. With a 14-day course of azithromycin and prompt initiation of dialysis, the patient’s pneumonia and systemic sequelae improved. This case demonstrates the importance of considering Legionella in the differential diagnosis of patients who present with community-acquired pneumonia and multi-organ dysfunction. Prompt diagnosis and management may decrease mortality associated with this disease sequela.

## Introduction

Legionella is one of the few bacteria, including Mycoplasma and Chlamydia, which causes atypical pneumonia. It presents with its unique range of extrapulmonary complications in a syndromic fashion [1]. The organ systems involved include gastrointestinal, musculoskeletal, neurological, and renal. The severity of the syndrome varies widely, and its association with acute renal failure due to rhabdomyolysis with concomitant neurologic dysfunction is rarely reported [1-3]. In this case report, we discuss an example of extrapulmonary Legionella with acute renal failure from rhabdomyolysis requiring dialysis in addition to encephalopathy and cerebellar dysfunction.

## Case presentation

An 80-year-old male with a past medical history of mild chronic obstructive pulmonary disease, well-controlled hypertension, gastroesophageal reflux disease, and prostate cancer status post radiation was brought to the emergency department by emergency medical services (EMS) due to altered mental status and shortness of breath for an unspecified period. His daughter called EMS after she could not get in touch with him for about two to three days. Prior to this admission, he lived alone and was able to perform all activities of daily living. When questioned in the hospital, his mental status returned to normal. He complained of flu-like symptoms for about a week in addition to fatigue, decreased appetite, fever, and cough. He had no recent trauma or exercise and denied chest pain, dizziness, nausea, vomiting, or bowel changes.

His family history was unremarkable. No history of tobacco, alcohol, or illicit drug use in the past was present. Home medications were simvastatin 20 mg, amlodipine 5 mg, ranitidine 150 mg, and budesonide nebulizer 0.5 mg/mL. There was no recent change in his medication list and no recent dosage changes.

On initial assessment, vitals were blood pressure of 130/78 mmHg, heart rate of 112 beats per minute and regular, oxygen saturation of 85%, which improved to 91% on 6 liters bi-nasal cannula, respiratory rate of 24 breaths per minute, and temperature of 101.3°F. The cardiac examination was only remarkable for tachycardia. The pulmonary examination revealed decreased vesicular breath sounds in the left lower lung field. Initial lab findings showed leukocytosis with increased lactic acid, creatinine, and creatinine kinase levels, as shown in Table 1.

**Table 1:** Initial laboratory results

Blood	Result	Reference range
Hemoglobin (g/dL)	15.1	12.0-16.0 (g/dL)
White blood cells (10*3u/L)	18.8	4.5-11.0 (10*3u/L)
Glucose (mg/dL)	169	70-99 (mg/dL)
Creatinine (mg/dL)	2.05	0.61-1.24 (mg/dL)
Sodium (mmol/L)	138	136-146 (mmol/L)
Calcium (mg/dL)	7.8	8.4-10.2 (mg/dL)
Potassium (mmol/L)	4.5	3.5-5.0 (mmol/L)
Phosphorus (mmol/L)	4.7	3-4.5 (mmol/L)
Bicarbonate (mmol/L)	19	7-18 (mmol/L)
Magnesium (mg/dL)	2.6	1.3-2.5 (mg/dL)
Thyroid-stimulating hormone (uIU/mL)	1.176	0.3-4.5 (uIU/mL)
Creatinine kinase	3,674	22-232 (iU/L)
Lactic acid	4.3	0.5-2.0 (mmol/L)
Aspartate aminotransferase	705	10-42 U/L
Alanine aminotransferase	190	10-60 U/L

**Figure 1:**
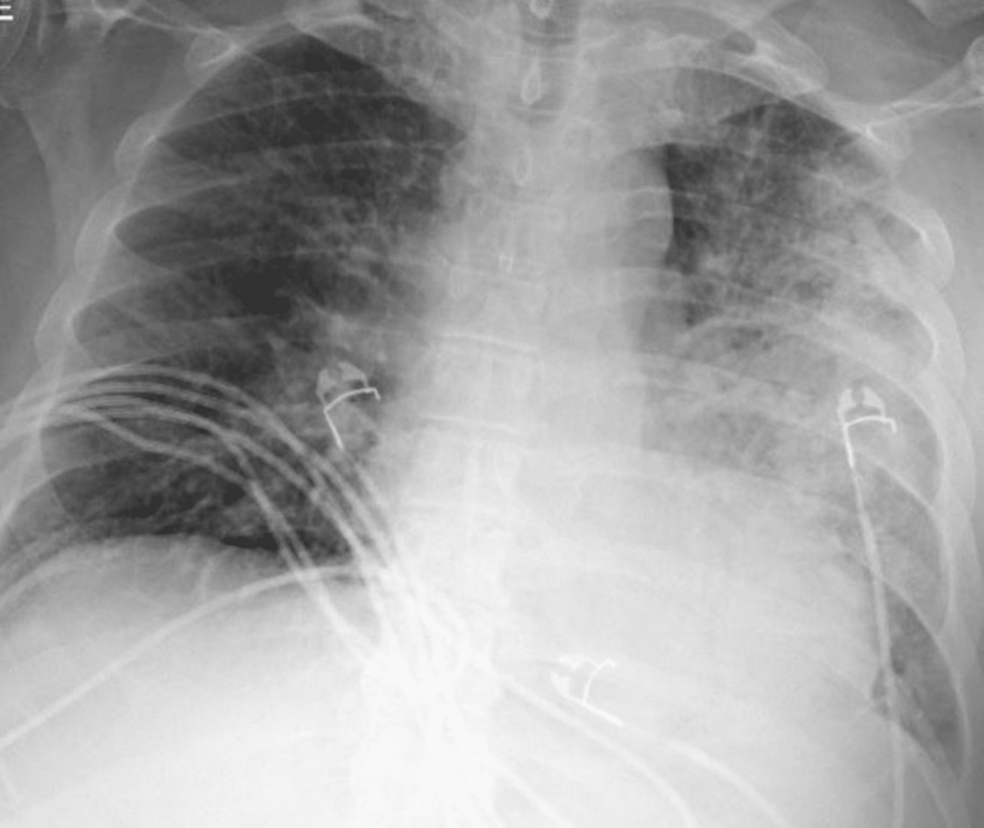
Initial chest X-ray

**Figure 2:**
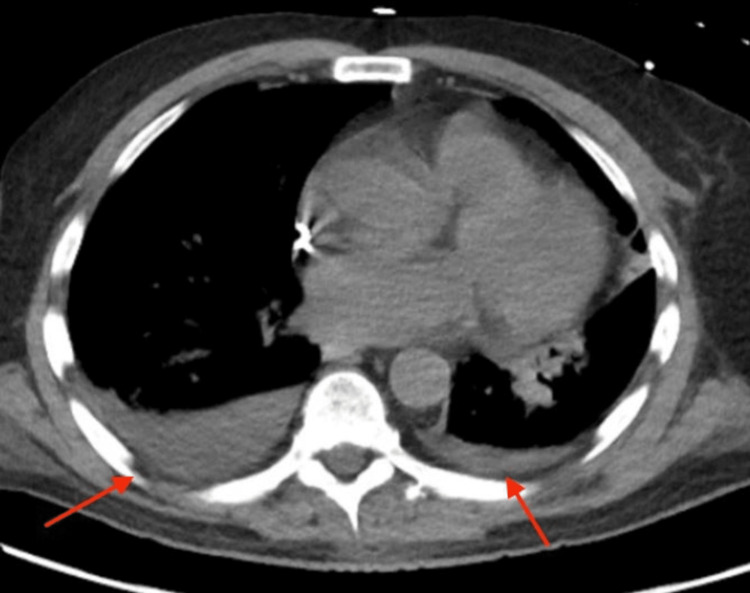
Computed tomography scan of the chest without contrast showing small bilateral pulmonary effusions (red arrows)

Chest X-ray revealed a left lower lobe infiltrate, and computed tomography of the chest without contrast revealed small bilateral pleural effusions (Figures 1, 2). He was initially treated for sepsis with intravenous fluids, vancomycin, piperacillin/tazobactam, and maintenance fluids. Legionella urine antigen came back positive on hospital day two, and antibiotics were switched to azithromycin. His home statin was held secondary to transaminitis.

On the third night of admission, the rapid response was called for hypotension and hypoxia. Laboratory results showed lactic acid of 4.9 mmol/L, creatine kinase of 17,495 iU/L, blood urea nitrogen (BUN) of 82 mg/dL, and creatinine of 4.99 mg/dL. The patient was not oliguric and urinalysis was positive for blood with no RBCs. Fluids were started and oxygen supplementation was increased to Optiflow (Fisher & Paykel Healthcare, Auckland, New Zealand).

Over the next few days, the patient's creatinine kinase levels began to trend down but the severe non-oliguric acute kidney injury and uremia continued to worsen. BUN/creatinine peaked at 151/7.31 mg/dL. The patient also developed confusion in addition to moderate to severe scanning dysarthria. Computed tomography of the head was performed and was negative for stroke or any other acute pathology. Given this altered mental status, the patient was placed on a nothing-by-mouth diet and the decision was made to start dialysis, as there was a concern that uremic encephalopathy was contributing to the patient’s neurologic status. Dialysis was continued through the rest of the hospital stay and a 14-day course of azithromycin was finished. The patient’s confusion gradually improved and the patient was weaned back to room air. The weaning was aided by two thoracenteses for a left-sided parapneumonic effusion. His dysarthria also significantly improved from moderate-severe to mild, but upon discharge planning, it was found that the patient had dysphagia and a moderately unsteady gait. A modified barium swallow showed mild oropharyngeal dysfunction. Liver function tests also returned to normal and the patient was discharged safely to rehab after a 17-day hospital stay. One month after discharge, physical therapy noted that the patient still had mild residual gait dysfunction.

## Discussion

Legionella accounts for only 2-9% of all cases of community-acquired pneumonia, but there has been a 192% increase in incidence from 2000 to 2009. Only 4% of cases are associated with a local outbreak [4]. Age, chronic lung disease, smoking, and immunocompromised states are all risk factors for the disease. Despite the relative rarity of Legionella, it is the second most common cause of pneumonia requiring intensive care unit admission and has a 42% mortality rate in community-acquired cases [2]. Diagnosis of Legionella is typically made with urine antigen testing, which has a sensitivity of 70-100% and specificity approaching 100% [5]. Preferred antibiotics are azithromycin or levofloxacin, which are bactericidal and have activity against all Legionella species. The duration of treatment is five to 14 days depending on the severity of the disease, response to treatment, and immune state of the patient [5].

Rhabdomyolysis is a known complication of Legionella. The mechanism of action is still unclear, but theories include the direct invasion of bacteria into the muscle and the release of bacterial endotoxins into the bloodstream leading to muscle damage. The majority of these cases cause severe acute kidney injury, which requires dialysis. A 2019 article reviewed 15 case reports on Legionella complicated by rhabdomyolysis. Of the 15 cases, 11 required dialysis, and one patient died before initiation of dialysis [6]. There is a useful score called the McMahon score, which risk stratifies patients with rhabdomyolysis. Scores of 6 or higher indicate higher risk and patients should receive renal protective therapies irrespective of creatinine kinase levels. Scores > 10 indicate the highest risk and our patient was in that group, which indicated our patient had a 52% chance of requiring renal replacement therapy or death [7]. Given the high percentage of Legionella rhabdomyolysis cases requiring dialysis, it is reasonable for these patients to have a lower threshold to initiate renal protective strategies. Further research is needed in this area. In the meantime, it is useful to utilize validated scoring systems like the McMahon score to risk-stratify patients.

Neurologic dysfunction is considered part of the classic syndrome of Legionnaire disease along with pneumonia, hyponatremia, and gastrointestinal symptoms. The most common presentation of neurologic dysfunction is encephalopathy. While our patient did have encephalopathy, he also had a constellation of findings that is most typical of the cerebellar motor syndrome, which has been estimated to occur in 3.7% of patients with Legionella [8]. The evidence of cerebellar motor syndrome in our patient included unsteady gait, scanning dysarthria, and oropharyngeal dysphagia. The scanning dysarthria is particularly characteristic of cerebellar dysfunction. It manifests with speech that is slurred with variable rhythm and force [9]. The complicated mechanism of swallowing provides a good example of the role the cerebellum plays in motor planning and coordination. In the swallowing mechanism, the cerebellum modulates the interplay between the cortex and brainstem to alter the strength and sequence of oropharyngeal muscle contraction and strength [10]. Given the extensive role of the cerebellum in normal motor function, it is important to recognize Legionella patients with signs of cerebellar dysfunction as they may require more intensive physical and speech therapy both during their hospital stay and past discharge. This is because there is evidence that cerebellar motor syndrome in Legionella patients may persist to some extent long after the resolution of other symptoms. A small case series found that 12 of 17 patients with long-term follow-up had persistent dysarthria and gait instability with the longest follow-up being three years [11].

The mechanism of neurologic dysfunction of Legionella is unknown and often there is no evidence of abnormal imaging or abnormal cerebral spinal fluid [11]. Current theories revolve around a possible neurotoxin or some sort of immune-mediated mechanism [3], the latter of which is supported by a small case series published in *JAMA Neurology* on acute disseminated encephalomyelitis in patients with Legionnaire disease (ADEM) [12]. ADEM in these patients was diagnosed via magnetic resonance imaging (MRI). The authors argue that many of the previous cases of Legionella with neurologic sequelae did not find evidence of abnormal imaging because there was a lack of utilization and availability of advanced imaging techniques such as MRI or single-photon emission computed tomography (SPECT). There has been some evidence that SPECT may be the superior imaging modality for neurologic sequelae of Legionella as there have been cases where SPECT has picked up prolonged cerebellar hypoperfusion not seen on MRI. This also provides evidence for the immune-mediated mechanism as this pattern seen on SPECT is similar to what has been seen on SPECT in CNS lupus patients [13]. Advanced imaging was not performed on our patient because his cerebellar dysfunction showed significant clinical improvement prior to discharge.

## Conclusions

In conclusion, our case is an example of the potential severe multifactorial organ dysfunction that can occur in Legionella. Legionella should be high on the differential for any patient with signs of community-acquired pneumonia and extrapulmonary disease. After diagnosis, risk stratification for severe renal impairment should be performed given the high rates of severe kidney injury requiring dialysis in those with rhabdomyolysis. Close monitoring of neurologic status both during and after the primary disease is also required as patients are at risk of developing severe neurologic sequelae. Increased utilization of advanced imaging in those with persistent, severe neurologic sequelae may provide more evidence that the pathophysiology of this dysfunction involves some form of immune response. While neurologic impairment often resolves with the completion of antibiotics, some patients like ours have some form of residual cerebellar findings, which may persist long after the acute phase of the syndrome. This signals a need for prolonged physical therapy and a strong patient support system.
